# Comparison of MR enteroclysis with video capsule endoscopy in the investigation of small-intestinal disease

**DOI:** 10.1007/s00261-012-9892-4

**Published:** 2012-04-18

**Authors:** Stijn J. B. Van Weyenberg, Koen Bouman, Maarten A. J. M. Jacobs, Brendan P. Halloran, Donald L. Van der Peet, Chris J. J. Mulder, Cornelis Van Kuijk, Jan Hein T. M. Van Waesberghe

**Affiliations:** 1 Department of Gastroenterology and Hepatology, VU University Medical Centre, De Boelelaan 1118, 1081 HV Amsterdam, The Netherlands; 2 Department of Gastroenterology and Hepatology, University of Alberta Hospital, Edmonton, AB Canada; 3 Department of Surgery, VU University Medical Centre, Amsterdam, The Netherlands; 4 Department of Radiology, VU University Medical Centre, Amsterdam, The Netherlands

**Keywords:** Small intestine, Intestinal neoplasms, Magnetic resonance imaging, Capsule endoscopy, Sensitivity and specificity

## Abstract

**Purpose:**

To evaluate the diagnostic accuracy of MR enteroclysis and to compare it to video capsule endoscopy (VCE) in the analysis of suspected small-bowel disease.

**Methods:**

We performed a retrospective analysis of 77 patients who underwent both MR enteroclysis and VCE and compared the findings of these studies with the findings of enteroscopy, surgery, or with the results of clinical follow-up lasting ≥2 years.

**Results:**

Findings included malignant neoplasms (*n* = 13), benign neoplasms (*n* = 10), refractory celiac disease (*n* = 4), Crohn’s disease (*n* = 2) and miscellaneous conditions (*n* = 10). Specificity of MR enteroclysis was higher than that of VCE (0.97 vs. 0.84, *P* = 0.047), whereas sensitivity was similar (0.79 vs. 0.74, *P* = 0.591). In 2/32 (6.3%) patients with both negative VCE and negative MR enteroclysis a positive diagnosis was established, compared to 5/11 (45.5%) patients in whom VCE was positive and MR enteroclysis was negative (likelihood ratio 8.1; *P* = 0.004), 9/11 (81.8%) patients in whom MR enteroclysis was positive and VCE was negative (likelihood ratio 23.5; *P* < 0.0001), and all 23 patients in whom both VCE and MR enteroclysis showed abnormalities (likelihood ratio 60.8; *P* < 0.0001).

**Conclusions:**

VCE and MR enteroclysis are complementary modalities. In our study-population, MR enteroclysis was more specific than VCE, while both produced the same sensitivity.

Advances in both radiological as endoscopic techniques have resulted in improved non-invasive diagnostic options for patients with suspected small-intestinal diseases including midgastrointestinal bleeding (MGIB), celiac disease, Crohn’s disease, malignant neoplasms and polyposis syndromes [1–7]. Radiological modalities for small bowel disease include small bowel follow through, double contrast barium-air enteroclysis, CT enterography or enteroclysis, and MR enterography or enteroclysis [8]. Direct non-invasive endoscopic visualization of the small intestine can be performed using video capsule endoscopy (VCE) [9, 10]. Images captured by this camera are transmitted to a receiver to be reviewed by a gastroenterologist.

In general, non-invasive radiological modalities and/or VCE are used to determine whether more invasive device-assisted enteroscopic techniques, such as double and single-balloon endoscopies (DBE and SBE) or spiral-assisted endoscopy, are needed, and to guide the route of insertion [8, 11, 12].

Several studies have compared the diagnostic yield of radiological modalities with VCE [13–19]. Of the radiological methods used to investigate the small bowel, MR enterography and enteroclysis are of particular interest, because the absence of ionizing radiation facilitates both the use in younger patients as well as repetitive use, which might be necessary in Crohn’s disease or small-intestinal polyposis syndromes. The place that these relatively novel procedures will occupy in the diagnostic algorithm of suspected small-intestinal conditions remains to be fully determined, especially since in most studies comparing radiological modalities and VCE, no reference standard was used. In addition, despite recent studies highlighting the diagnostic accuracy of MR enteroclysis in patients with suspected small bowel neoplasms and in patients with suspected refractory celiac disease, there are no studies comparing the diagnostic value of MR enteroclysis and VCE [20–22].

Therefore, we aimed at evaluating the diagnostic accuracy of MR enteroclysis in patients with suspected small-intestinal disease, and to compare this with VCE.

## Methods

### Study population

From the records of the departments of gastroenterology and radiology, we identified 98 patients who had undergone both VCE and MR enteroclysis between June 2004 and January 2009. These comprised 98 (17.9%) of all 546 MR enteroclysis studies performed in this period, and 98 (9.6%) of all 1012 VCE studies performed in this period. We excluded seven patients who had surgery (*n* = 1), chemotherapy or anti-inflammatory therapy (*n* = 5), or underwent an endoscopic intervention in the small intestine using DBE (*n* = 1) in between the two studies. In addition, we excluded 12 patients who had not undergone any invasive reference test, and in whom clinical follow-up was less than 24 months. Two patients were not included because of insufficient data. The total group comprised 77 patients (age range 4–87 years; mean 51 years; median 56 years). There were 35 female patients (age range 11–87 years; mean 48 years; median 48 years) and 42 male patients (age range 4–83 years; mean 53 years; median 58 years). Clinical data were retrieved from medical charts and included patient demographic data, both the indication for small bowel investigation and the specific indication for each modality, the order of the examinations, any complications, the duration of follow up and the clinical outcome. All the patients had undergone esophagogastroduodenoscopy and ileocolonoscopy at least once before VCE and MR enteroclysis were performed.

### Video capsule endoscopy

All VCE studies were performed using either the Given Pill cam SB system (Given imaging, Yoqneam, Israel) or the Mirocam system (Intromedic, Seoul, Korea). All the patients received two litres of polyethylene glycol solution (Klean Prep, Norgine, Amsterdam, The Netherlands) at midday, 1 day before the examination and nil by mouth after midnight before the examination. The capsule was ingested with a small amount of water. Patients were allowed liquids 4 h after ingestion of the capsule solid food after 8 h.

### MR enteroclysis

After an overnight fast, a 9-French nasojejunal tube (Hospimed International, Zwolle, The Netherlands) was positioned distal to the duodenojejunal junction with fluoroscopic guidance. Next, during MR imaging, a minimum of 2000 ml 0.5% methylcellulose solution in water was infused through the tube, at a flow rate of 80–100 ml/min, using a MR-compatible infusion pump system (Watson Marlow, Falmouth, United Kingdom).

We performed 1.5-T MR imaging (Sonata; Siemens Medical Systems, Erlangen, Germany) using a 16-element-phased array surface coil. Gradient strength was 40 mT with a maximal gradient slope of 200 mT/ms. The imaging protocol consisted of multiple axial and coronal breath-hold true fast imaging with steady-state precession (FISP) sequences (repetition time / echo time: 4.3/2.2 msec; flip angle 70°; section thickness 4 mm; intersection gap 0.8 mm, field of view 320–400 mm; matrix 288 × 512) in multiple breath-hold series, to cover whole the abdomen. In between the true-FISP sequences, a heavily T2-weighted half-Fourier acquisition single-shot fast spin-echo (HASTE) sequence (repetition time/echo time: 1000/90 msec; echo train length 224; flip angle 150°; section thickness 6 mm; intersection gap 3 mm, field of view 320–400 mm; matrix 288 × 512) was performed three times with full abdominal coverage to follow infusion of the contrast agent. Images were acquired with patients in the prone position, to reduce the abdominopelvic volume. Acquisition time per series was 20–25 sec. All series were repeated at least five times in a row. Imaging was stopped when optimal distensions of the full small-bowel and cecum were obtained. The total imaging time per patient was approximately 30 min. No intravenous contrast material was used. Because of the short acquisition time of the true-FISP sequence and the enteroclysis-related atonia of the small intestine, no antispasmodics were administered. This protocol was used during the entire study period.

### Data analysis

All capsule studies were reviewed in clinical practice by one of two gastroenterologists experienced with VCE, or by a senior fellow directly supervised by one of these gastroenterologists. A positive VCE-diagnosis was defined as the presence of one or more lesions with a high potential of causing the patients symptoms or allowing a likely diagnosis, e.g., angiodysplasia, multiple ulcers, stenosis, polyps or tumors. Lesions of unknown significance, such as isolated erosions or red spots, were not considered to be positive findings. Additional data collected included the location of abnormalities encountered, type of abnormalities encountered and whether the capsule had reached the cecum within battery lifetime. Quality of bowel preparation was scored as good, moderate or poor.

All MR-studies were interpreted in clinical practice by one of two gastrointestinal radiologists. A positive MR-diagnosis was defined as the presence of any abnormality with a high potential of causing the patient’s symptoms or allowing a likely diagnosis, such as stenosis, polyps or tumors or findings considered diagnostic for refractory celiac disease or Crohn’s disease [2, 20]. The quality of bowel distension was scored as good, moderate or poor. All studies fulfilling the entry-criteria were included in the final analysis, in analogy to an intention-to-treat protocol. Therefore, incomplete capsule studies or failed enteroclysis studies were not excluded

### Reference standard

As a standard of reference for the presence of abnormalities, we used (**a**) histopathology findings (*n* = 41) obtained via biopsy specimens collected during DBE (*n* = 29) and / or surgical resection (*n* = 12); (**b**) (the absence of) endoscopic findings at DBE without histopathological confirmation (*n* = 16).

If no DBE or surgery was performed, then the results of clinical follow-up lasting at least 24 months (*n* = 20; mean follow-up duration, 40 months; range, 24–68 months) were used as standard of reference. DBE was performed according to the method described in detail by Yamamoto et al. [23]. In general, the route of insertion (either peroral or peranal) was dictated by the findings of MR enteroclysis and VCE [24].

### Statistical analysis

We compared qualitative variables with the Fisher exact test or Chi-square test. Quantitative variables were compared with the two-sided Student *t* test. The sensitivity, specificity, negative predictive value, positive predictive value and overall accuracy of MR enteroclysis and VCE were calculated and compared by using the Fisher exact test. *P* *<* 0.05 was considered to indicate a statistically significant difference.

## Results

### Details of capsule studies and MR enteroclysis studies

Indications for the capsule studies and MR enteroclysis studies are shown in Table 1. Of the 77 patients included, 61 underwent VCE as the first, and MR enteroclysis as the second small-bowel investigation. The order in which VCE and MR enteroclysis was performed was not associated with sex, age, main indication for investigation of the small bowel or the standard of reference.

**Table 1:** Details on the study population according to the order of diagnostic tests

Parameter	Capsule endoscopy first	MR enteroclysis first	Total study group	*P* value
Number of patients, *n*	61	16	77	
Female/male, *n*	28/33	7/9	35/42	0.878^a^
Mean age, *y* (SD)	51 (20)	50 (19)	51 (19)	0.887^b^
Main indication, *n* (%)				0.180^c^
Suspected MGIB	30 (49.2)	4 (25.0)	34 (44.2)	
Polyposis syndrome	10 (16.4)	4 (25.0)	14 (18.2)	
Suspected refractory celiac disease	10 (16.4)	1 (6.3)	11 (14.3)	
Abdominal pain	5 (8.2)	2 (12.5)	7 (9.1)	
Malabsorption	3 (4.9)	3 (18.8)	6 (7.8)	
Crohn’s disease	3 (4.9)	2 (12.5)	5 (6.5)	
Mean duration of clinical follow-up, *y* (SD)	42 (17)	38 (16)	40 (17)	0.476^b^

MGIB, midgastrointestinal bleeding; DBE, double-balloon endoscopy

^a^ Calculated with the two-sided Student *t* test

^b^ Calculated with Fishers exact test

^c^ Calculated with the Chi-square test

In eight (13.1%) of the 61 patients who underwent VCE first, MR enteroclysis was ordered because of either insufficient bowel preparation or incomplete visualization of the small intestine limited the diagnostic quality of the capsule study In 1 (6.3%) of the 16 patients who underwent MRE first; subsequently, VCE was performed because of impaired quality of the MR-study. This difference was not statistically significant (*P* = 0.675). In 19 (24.7%) of the 77 capsule studies, the capsule failed to reach the colon within the battery’s lifespan. The quality of VCE-examinations was considered good in 55 (71.4%) of the 77 patients, whereas the quality of MR enteroclysis was considered good in 65 (84.4%) of the 77 patients. This trend failed to reach statistical significance (*P* = 0.052). Five (6.5%) of the 77 intended MR enteroclysis studies were eventually performed without a nasojejunal tube, because of intolerance to the tube (*n* = 3) or failed placement due to large hiatal hernia (*n* = 2). Two of these studies were considered of poor quality, whereas in the other three studies, sufficient oral contrast could be delivered to achieve moderate bowel distension.

**Fig. 1:**
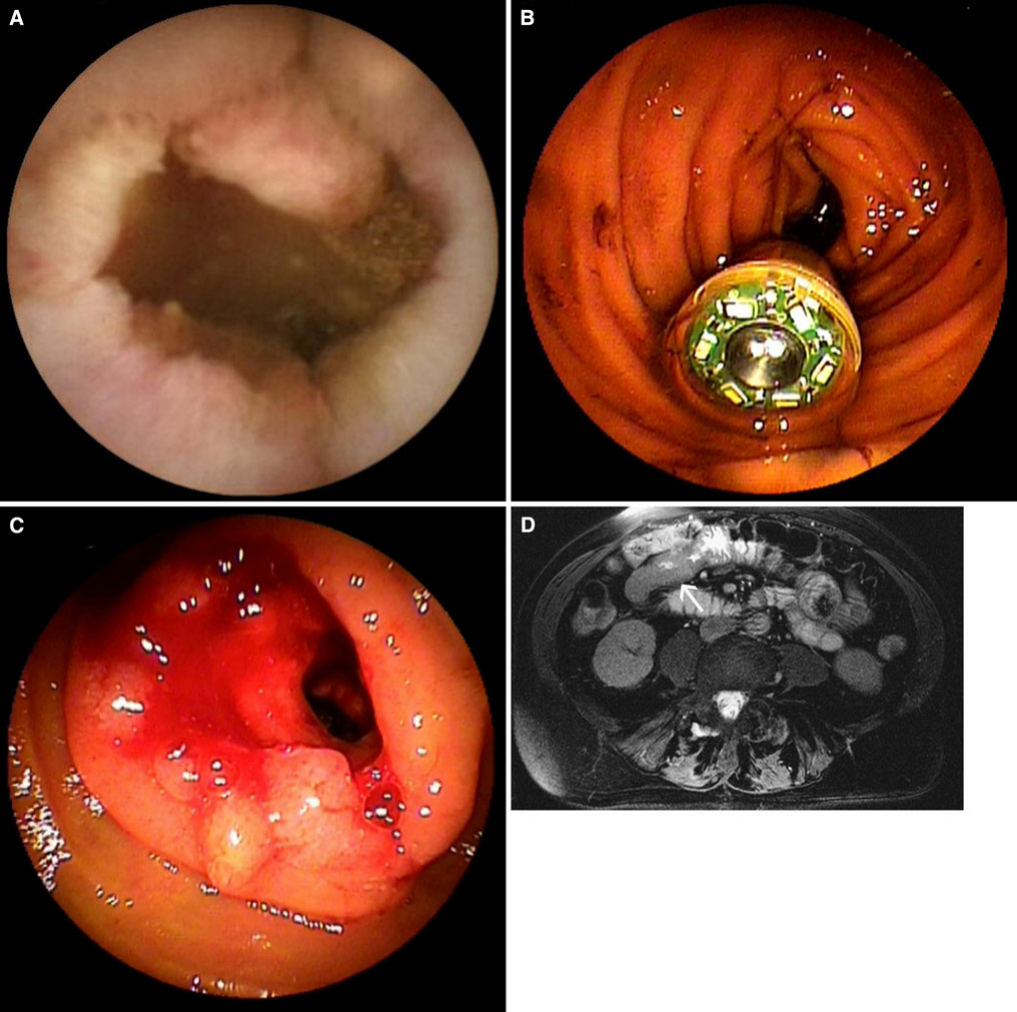
82-Year-old female with iron-deficiency and negative conventional bidirectional endoscopy. **A** Capsule image showing an irregular stenotic mass lesion. **B** Emergency double-balloon endoscopy was performed because of obstructive symptoms 6 h after ingestion of the capsule and showed the capsule in the proximal small intestine. **C** After endoscopic removal of the capsule, a stenotic mass lesion became visible. Biopsy specimens revealed the lesion to be carcinoma. **D** Transverse True FISP MR enteroclysis image showing wall thickening and obstruction of the proximal jejunal lumen (*arrow*).

In two patients, symptomatic retention of the capsule occurred, requiring urgent retrieval by emergency DBE. Both cases of retention were caused by stenotic small-intestinal cancers and occurred in patients in whom VCE was performed before MR enteroclysis (Fig. 1). Vomiting during the MR enteroclysis procedure occurred in four patients, impairing the quality of the examination in two patients. No other complications of MR enteroclysis occurred.

### Findings

**Fig. 2:**
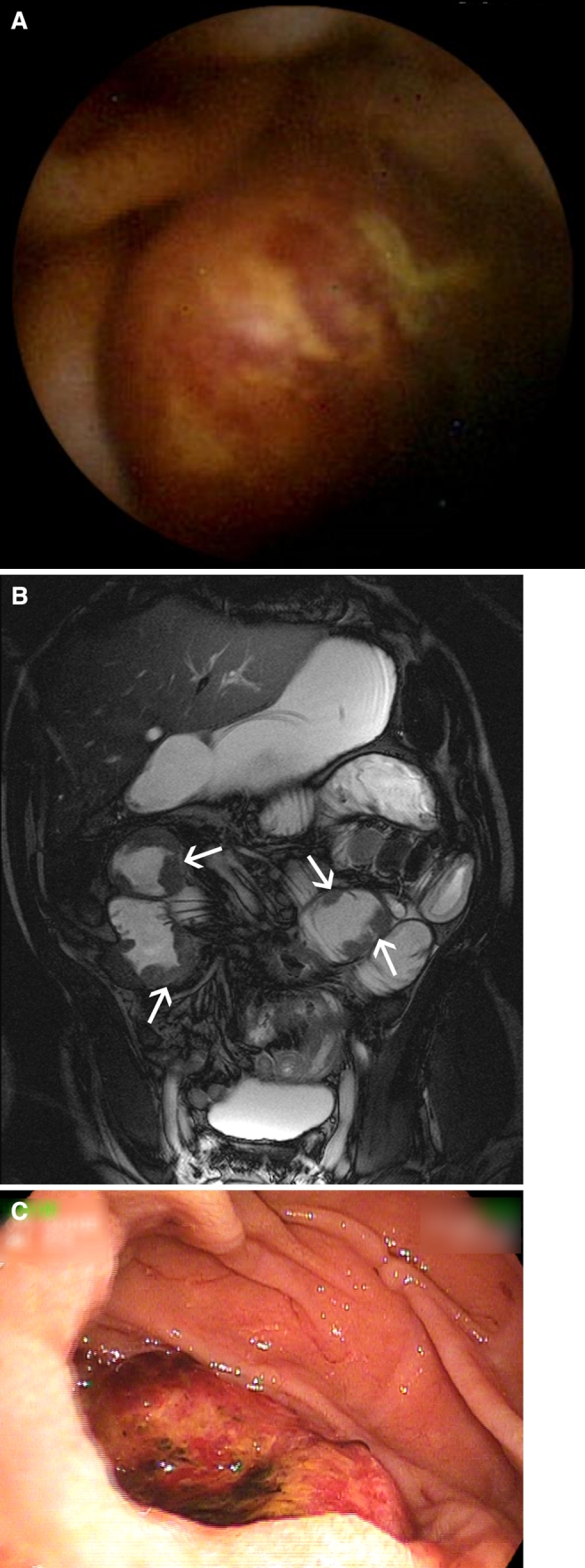
61-Year-old male patient with iron deficiency and negative conventional bi-directional endoscopy. **A** Capsule image showing a round ulcerative lesion in the proximal small-bowel. **B** Coronal True-FISP MR enteroclysis image showing multiple mass lesions in the proximal jejunum (*arrows*). **C** Double-balloon endoscopy images showing an ulcerating mass in the proximal jejunum. Biopsy specimens showed this lesion to be a large-cell B-cell lymphoma.

**Fig. 3:**
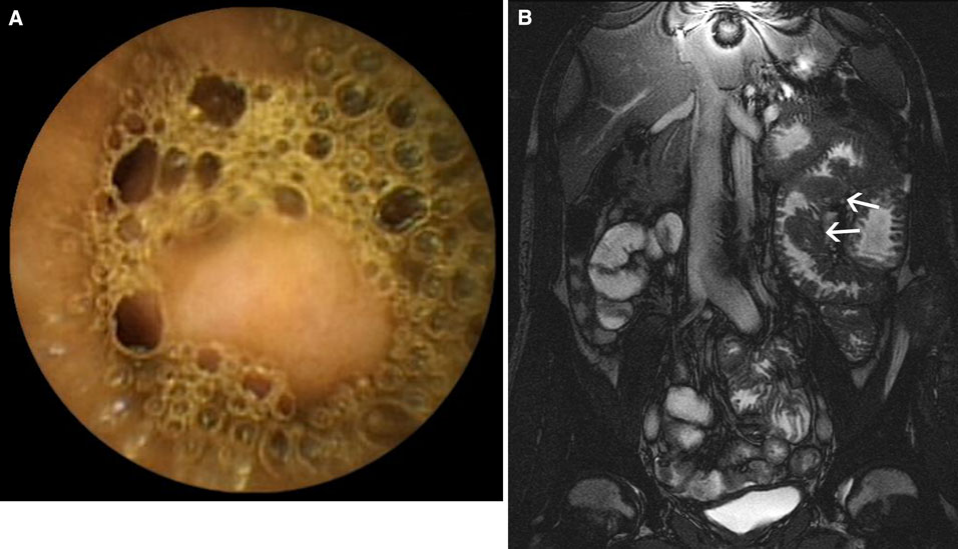
50-Year-old female with non-small-cell lung cancer and overt midgastrointestinal bleeding. **A** Capsule image showing a smooth intraluminal mass in the centre of the image. **B** Coronal True-FISP MR enteroclysis image showing multiple mass lesions in the proximal ileum (*arrows*).

**Fig. 4:**
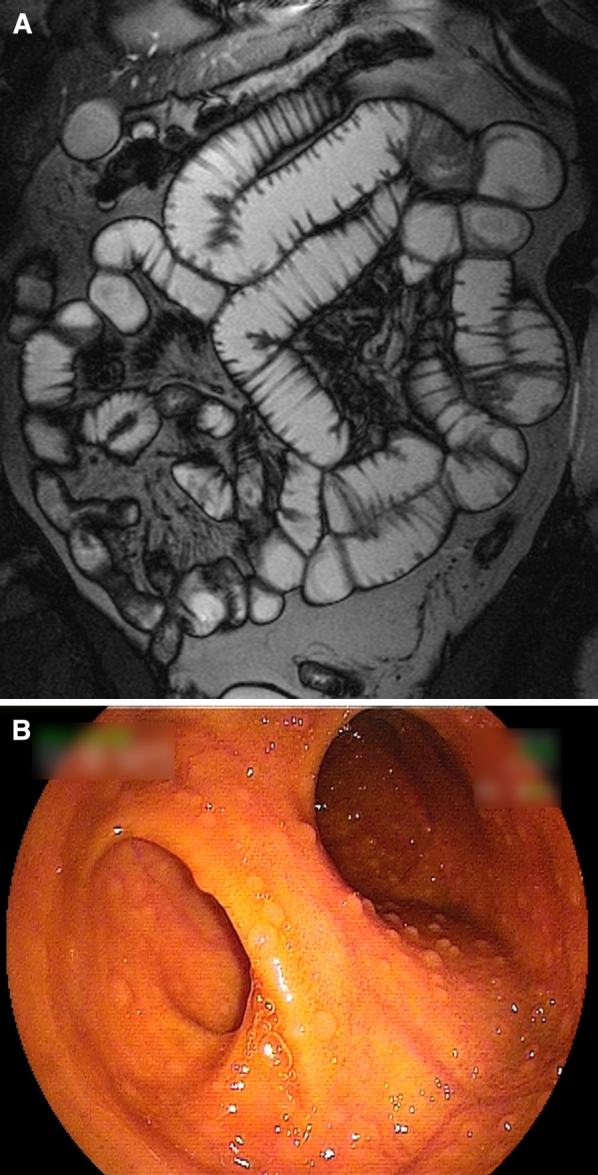
37-Year-old male patient with iron deficiency anemia and negative conventional bi-directional endoscopy. **A** Coronal True-FISP MR enteroclysis image showing slight infiltration of the mesenteric fat, without other abnormalities. **B** Double-balloon endoscopy was performed after capsule endoscopy (not shown) failed to show any abnormalities. 40 cm Proximal to the ileocecal valve, a diverticulum (*left ostium in the image*) was observed. After surgical resection of this Meckel’s diverticulum, the anemia was resolved.

Overall, MR enteroclysis and VCE were both negative in 32 (41.6%) patients and both positive in 23 (29.9%) patients, resulting in an agreement in 55 (71.4%) patients (Figs. 2, 3). In 11 (14.3%) patients, VCE was positive and MR enteroclysis negative, whereas in another group of 11 (14.3%) patients, MR enteroclysis was positive and VCE negative. A positive diagnosis was established by means of the reference tests or during >2 years of clinical follow-up in 39 (50.6%) of 77 patients (Table 2). In 2 of the 32 (6.3%) patients with both negative VCE and negative MR enteroclysis, a positive diagnosis was established. In comparison, a positive diagnosis was established in 5 of the 11 (45.5%) patients in whom VCE was positive and MR enteroclysis was negative (likelihood ratio 8.1; *P* = 0.004); in 9 of the 11 (81.8%) patients in whom MR enteroclysis was positive and VCE was negative (likelihood ratio 23.5; *P* < 0.0001); and in all, 23 patients in whom both VCE and MR enteroclysis showed abnormalities (likelihood ratio 60.8; *P* < 0.0001) (Fig. 4).

**Table 2:** Details of final diagnosis according to the different reference tests used and in the complete group

Diagnosis	Reference standard				Total group (*n* = 77)
	DBE with histology (*n* = 29)	Surgery with histology (*n* = 12)	DBE without histology (*n* = 16)	Clinical follow-up >2 years (*n* = 20)	
Malignant neoplasms	4	9	0	0	13
Carcinoma		5			4
Lymphoma	3	1			4
Metastasis	1	2			3
Neuro-endocrine tumor		1			1
Benign neoplasms	6	3	0	1	10
Peutz–Jeghers syndrome	4	1		1	6
Cowden syndrome		1			1
Sporadic adenoma	1				1
Inflammatory fibroid polyp		1			1
Lipoma	1				1
Refractory celiac disease	3	0	0	1	4
Crohn’s disease	2	0	0	0	2
Vascular malformations	0	0	1	0	1
Other conditions	4	0	4	1	9
Meckel’s diverticulum	1		1		2
NSAID-related stenosis			1		1
Small-intestinal diverticulitis			1		1
Auto-immune enteropathy				1	1
Post-surgical stenosis			1		1
Eosinophilic enteritis	1				1
Lymphangiectasia	1				1
Whipple’s disease	1				1
Negative diagnosis	10	0	11	17	38

*Data* are number of patients

DBE, double-balloon endoscopy

Diagnostic accuracies of both MR enteroclysis and VCE are shown in Table 3. Except for the overall specificity, which was better for MR enteroclysis than for VCE, the test characteristics of both modalities did not differ significantly. No clear relation between the indication for small-intestinal analysis and differences between test characteristics of both VCE and MR enteroclysis was observed (data not shown).

**Table 3:** Performance characteristics of video capsule endoscopy and MR enteroclysis

Parameter	Video capsule endoscopy	MR enteroclysis	*P* value
Patients with true positive finding, *n* (%)	29 (37.7)	31 (40.3)	
Patients with true negative finding, *n* (%)	32 (41.6)	37 (48.1)	
Patients with false positive finding, *n* (%)	6 (7.8)	1 (1.3)	
Patients with false negative finding, *n* (%)	10 (13.0)	8 (10.4)	
Sensitivity (95% CI)	0.74 (0.58–0.86)	0.79 (0.63–0.90)	0.591
Specificity (95% CI)	0.84 (0.68–0.93)	0.97 (0.85–1.00)	0.047
Positive predictive value (95% CI)	0.83 (0.66–0.93)	0.97 (0.82–1.00)	0.061
Negative predictive value (95% CI)	0.76 (0.60–0.87)	0.82 (0.67–0.91)	0.488
Overall accuracy (95% CI)	0.79 (0.69–0.87)	0.88 (0.79–0.94)	0.126

CI, confidence interval

Table 4 summarizes the diagnostic accuracies of VCE and MR enteroclysis according to final diagnosis, as established with the references tests, or after 2 years of clinical follow-up. Both MR enteroclysis and VCE failed to detect a Meckel’s diverticulum and a case of Whipple’s disease. Of the 26 patients with either benign or malignant neoplasms, VCE failed to detect these in five patients. The neoplasms not detected by VCE were benign in three patients (small Peutz–Jeghers polyps, *n* = 1; sporadic adenoma, *n* = 1; lipoma, *n* = 1) and malignant in two patients (neuro-endocrine tumor, *n* = 1; ileocolonic carcinoma, *n* = 1). MR enteroclysis failed to detect one small hamartoma in a patient with Peutz–Jeghers syndrome. This patient did not tolerate the nasojejunal tube and underwent enterography instead of enteroclysis.

**Table 4:** Diagnostic accuracies of video capsule endoscopy and MR enteroclysis according to final diagnosis

Final diagnosis and modality	True positive	False negative	Total
Malignant neoplasms			
VCE	11 (84.6)	2 (15.4)	13
MR enteroclysis	13 (100.0)	0 (0.0)	13
Benign neoplasms			
VCE	7 (70.0)	3 (30.0)	10
MR enteroclysis	9 (90.0)	1 (10.0)	10
Refractory celiac disease			
VCE	4 (100.0)	0 (0.0)	4
MR enteroclysis	3 (75.0)	1 (25.0)	4
Crohn’s disease			
VCE	0 (0.0)	2 (100.0)	2
MR enteroclysis	2 (100.0)	0 (0.0)	2
Vascular malformations			
VCE	1 (100.0)	0 (0.0)	1
MR enteroclysis	0 (0.0)	1 (100.0)	1
Other conditions			
VCE	6 (66.7)	3 (33.3)	9
MR enteroclysis	4 (44.4)	5 (55.6)	9

All *data* are number of patients, with *percentages* between *parentheses*

VCE, video capsule endoscopy

Regarding non-neoplastic lesions, MR enteroclysis was false-negative and VCE true positive in a patient with a flat vascular malformation, a patient with ulcerative jejunitis, a patient with auto-immune enteropathy, a patients with a Meckel’s diverticulum and a patient with a short NSAID-related stenosis. MR enteroclysis was true positive and VCE false negative in two patients with Crohn’s disease and one patient with a stenotic jejunual surgical anastomosis. In all these three patients, VCE was incomplete.

**Fig. 5:**
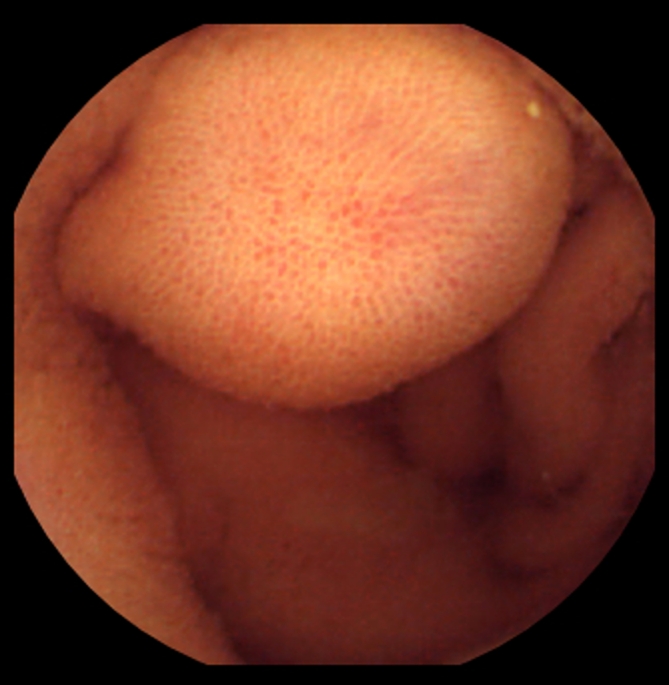
80-Year-old male patient with suspected midgastrointestinal bleeding. Video capsule endoscopy image showing a bulge falsely interpreted as submucosal mass. Further analysis with MR enteroclysis and double-balloon endoscopy could not confirm the presence of this suspected lesion.

In seven patients either MR enteroclysis (*n* = 1) or VCE (*n* = 6) detected abnormalities that could not be confirmed with the reference test. Therefore, these findings were considered to be false positive. In one patient, MR enteroclysis detected inflammation of the distal ileum, which could not be confirmed with DBE. VCE did not reveal any abnormalities in that patient. In three patients, distal ulcerative lesions were not confirmed with DBE. In two patients, VCE seemed to depict submucosal lesions that were not found at DBE (Fig. 5). In one patient, VCE detected probable stenotic intestinal segments, which were not identified during DBE. In none of these patients did MR enteroclysis show any abnormalities.

## Discussion

We performed a direct comparison of VCE and MR enteroclysis in 77 patients with suspected small-intestinal disease, and related the findings to a reference test to determine the diagnostic performance of both modalities. The specificity of MR enteroclysis was higher than that of VCE, but all other performance characteristics were similar. VCE and MR enteroclysis are complementary techniques that can be used to confirm either positive or negative single-study findings, or to further investigate the patients suspected of intestinal disease, despite negative single-study findings.

In light of the rarity of small-intestinal conditions, studies on the diagnosis of small-intestinal diseases inevitably result in relatively small, heterogeneous study populations, usually from tertiary referral centres. However, several aspects of our study can aid the diagnostic management of suspected small-intestinal conditions. First of all, five of the ten false-negatives of VCE concerned patients with small-intestinal neoplasms, two of which were malignant. All these tumors were recognized on MR enteroclysis. In our opinion, it is advisable to perform additional cross-sectional imaging in patients with suspected small-bowel neoplasms despite negative VCE [25]. A second concern with VCE regarding neoplasms is the difficulty to discriminate submucosal masses from bulges, which resulted in two false-positive VCE-studies in our series. Therefore, we think decisions concerning the management of suspected small-intestinal masses should not be made based on capsule findings alone [14]. There are several explanations for the possible superiority of MR enteroclysis over VCE in the detection of neoplasms: First, MR enteroclysis images the distended small intestine, while VCE, in contrast to conventional endoscopy, images the non-distended bowel. This can lead to both false-negative as well as false-positive VCE-findings. Secondly, sometimes the capsule moves very quickly to parts of the small-intestine. In combination with the unidirectional view of the capsule, this might lead to lesions being missed.

In almost 25% of the VCE studies performed, the capsule study was judged incomplete, which is comparable to other studies concerning VCE [26, 27]. This may limit the use of VCE as a surveillance tool in patients with polyposis syndromes. In two of our patients, symptomatic capsule retention occurred. None of these patients had any symptoms suggestive of small-intestinal stenosis, and both were diagnosed with stenotic small-bowel cancer. In case of suspected small-intestinal cancer or symptoms suggestive of small-intestinal stenosis, it might be advisable to perform MR enteroclysis as the initial modality [28, 29].

Many studies have compared VCE with radiological modalities in the diagnosis of Crohn’s disease [13, 30–38]. Unfortunately, most studies comparing VCE with radiological imaging suffer from two important flaws. First, patients in whom a stenosis is detected by a radiological modality are usually excluded from further analysis because VCE is not safe in these conditions, leaving only a subgroup of patients with mainly superficial inflammation in the final comparison. As can be suspected, superficial mucosal erosions and ulcers are better detected by VCE than by radiological imaging, resulting in superior results of VCE. It is doubtful whether such exclusion policies result in study populations representative of daily practice. A second important flaw is that most studies on the diagnosis of small-intestinal Crohn’s disease lack a reference test. Therefore, it is not possible to say whether all lesions detected by VCE are true-positive lesions, let alone whether they really are caused by Crohn’s disease. Since our study included only five patients suspected of Crohn’s disease, of whom only two eventually were diagnosed with Crohn’s disease, no conclusions on this subject can be drawn from our series.

Only a few studies have compared VCE with radiological modalities in populations not entirely composed of patients suspected of or established with Crohn’s disease. Rajesh et al. [39] compared the yield of VCE with that of either CT enteroclysis or fluoroscopic barium methylcellulose or carbon dioxide enteroclysis, and concluded that all modalities, except for barium methylcellulose enteroclysis, had similar diagnostic yield. Despite the limited number of patients per modality and the lack of a reference test, it was clear that VCE was superior in the detection of angioectasia. Khalife et al. [14] compared CT enteroclysis with VCE in 32 patients with obscure gastrointestinal bleeding, and concluded that the overall diagnostic yields were similar. As in our series, VCE seemed to perform less in patients with neoplasms, but better in patients with angioectasia. A study from Germany authored by Bocker et al. [18], compared MR enterography with VCE in 46 patients, and found MR enterography to be superior in patients with Crohn’s disease or obscure gastrointestinal bleeding. However, in the absence of a reference test, it is difficult to establish whether all positive findings were true-positive findings, which is especially important when subjective parameters like mucosal redness are being scored as positive findings. Since none of the included patients had a small-intestinal neoplasm, this aspect cannot be compared with our series. In general, midgastrointestinal bleeding is the most frequent indication for small-intestinal analysis [9, 40].

It is reasonable to assume that direct endoscopic assessment of the mucosa is a more reliable method to detect flat angioectasia, which are the most common cause for midgastrointestinal bleeding, than any radiological method available. On the other hand, there are several reasons as to why radiological imaging of the small intestine might be preferable in the detection of small-intestinal neoplasms: better estimation of size, number and location of lesions; no risk of capsule retention; assessment of extraluminal disease; and possibly superior sensitivity and specificity [14, 21, 22, 41]. Therefore, the index of suspicion of the referring physician usually dictates which modality is chosen.

Our study is limited by its retrospective nature, which inevitably has resulted in a selection bias. In the majority of patients evaluated at our departments, only a single study is performed. In general, we perform VCE in case of (suspected) obscure gastrointestinal blood loss, and prefer MR enteroclysis as the initial investigation of patients with (suspected) small-intestinal neoplasms. Only rarely do we perform both tests. This has resulted in a study group composed of patients with probably more complicated and rare small-intestinal conditions than most patients referred for VCE or MR enteroclysis. For instance, only one of the patients included in this study had angiodysplasia, while this is the most common lesion encountered in our population referred for VCE. Verification bias may further limit the generalizability of our results, since DBE and/or surgery were more frequently performed in patients with abnormal VCE and/or MR enteroclysis results. Since we have used the original interpretation of both VCE-studies as MR enteroclysis studies, interobserver agreement was not studied. Our MR-protocol did not include contrast-enhanced sequences. Recent studies showed that a MR enteroclysis protocol without contrast-enhancement had similar accuracy for the detection of small-intestinal neoplasms as a protocol including contrast-enhancement. The role of intravenous contrast in the detection of minute angioectasia is not clear [21, 22].

In conclusion, in our study population, the specificity of MR enteroclysis was significant higher than that of VCE, but all other performance characteristics were similar. VCE and MR enteroclysis can both be used to confirm negative or positive single-study findings. In addition, both modalities can be used to further investigate patients with a high clinical suspicion of having small-intestinal disorders, despite negative single-study findings. Further studies are required to prospectively investigate the optimal diagnostic algorithm for patients suspected of small-intestinal conditions. Such studies should also include whether certain patient characteristics, signs, or symptoms can be used to select the order in which small-intestinal investigations should be performed.
